# Care seeking behaviour and barriers to accessing services for sexually transmitted infections among female sex workers in Laos: a cross-sectional study

**DOI:** 10.1186/1472-6963-12-37

**Published:** 2012-02-14

**Authors:** Ketkesone Phrasisombath, Sarah Thomsen, Vanphanom Sychareun, Elisabeth Faxelid

**Affiliations:** 1 Faculty of Postgraduate Studies, University of Health Sciences, Vientiane, P.O Box 7444, Lao PDR; 2 Division of Global Health (IHCAR), Department of Public Health Sciences, Karolinska Institutet, Nobels vag 9 SE-171 77 Stockholm, Sweden

**Keywords:** STI, Care seeking behaviour, Female sex worker (FSW), Laos

## Abstract

**Background:**

Prompt, correct diagnosis and treatment with health information are essential components of reproductive tract infection (RTI) and sexually transmitted infection (STI) services. This study aims to describe care seeking behaviour and barriers to accessing RTI/STI services among female sex workers (FSWs) in Laos.

**Methods:**

A cross-sectional survey using closed and open-ended questions was performed in six districts along Road 9, traversing Savannakhet province from Thailand to Vietnam. In total, 407 FSWs were interviewed. The data were analyzed and presented descriptively. Multiple logistic regression analysis was applied to assess associations between respondents' background characteristics and care seeking behaviour.

**Results:**

About half of the respondents (49%) were less than or equal to 19 years of age, and 50% had started or completed secondary school. Fifty-eight percent had been engaged in sex work for less than 1 year. Eighty-six percent of the respondents reported RTI/STI signs or symptoms currently or in the last 3 months but only two-thirds of those with symptoms sought treatment. Source of treatment for the last RTI/STI episode was the drop-in centre (53%) followed by a public hospital (23%), private clinic (12%), private pharmacy (9%), and herbalist (2%). The main barriers to service use were long waiting time, inconvenient location of the clinic, not knowing where to get the services needed, and negative attitudes among healthcare providers. Care seeking behaviour was associated with longer duration of sex work (OR = 2.6, 95%CI 1.52-5.36). Forty-four percent received health information from peer educators, 34% from fellow friends, 26% from a pimp, and 26% had received information from a healthcare provider during the visit.

**Conclusion:**

There were several barriers to accessing RTI/STI services and they were related to both structural and individual factors. Innovative STI service strategies to inform FSWs about the importance of early diagnosis and treatment should be established. Continuous training for STI service providers focusing on counseling skills and awareness of the sexual health care needs for FSWs is recommended in order to minimize the barriers experienced by FSWs in this particular setting.

## Background

Laos, with a population of 5.6 million, is classified as a low HIV prevalence country with 0.2% of people aged 15-49 estimated to be HIV positive [1]. Sexually transmitted infections (STIs) represent a public health problem in Laos. In 2004, an HIV prevalence of 3.9% was detected in female sex workers (FSWs), whereas infection rates of Chlamydia and gonorrhoea were 33 and 18% respectively [2]. In 2005, the Government of Laos initiated a national strategic and action plan on HIV/AIDS/STIs aimed at expanding the national capacity for universal access to treatment, care and support to all in need [3]. The target groups included FSWs, mobile populations and drug users. Moreover, information and media campaigns directed at high risk groups and the general population have been launched throughout the country.

Due to economic reforms in recent decades, Laos has experienced economic expansion, increased business transactions, and an increasing tourist industry. Entertainment establishments have become a booming business in the big cities. However, these changes have also brought domestic and cross-border migration, which has increased social inequalities between people in rural and urban areas [4]. Problems related to poverty have also arisen due to the economic expansion. Thus, women have fewer opportunities for employment and are financially vulnerable, forcing some women to engage in commercial sex as a mean of survival (Phrasisombath K, Thomsen S, Sychareun V, Faxelid E: 'Health is Wealth and Wealth is health' -perception of health and ill-health among female sex workers in Savannakhet province (submitted)).

Despite economic development, Lao traditional culture and norms remain conservative. Sexuality is a sensitive issue and sexual relations outside marriage are strongly discouraged for women, especially among healthcare providers (HCPs) [5]. In Laos, sex work is considered immoral and previously FSWs were put in rehabilitation camps called Don Nang Island 'women island' due to inappropriate behaviour [6]. This is, however, no longer practiced. Commercial sex is still illegal, although a visible phenomenon in Lao society. There are two types of FSWs in Laos, including street-based FSWs and venue-based FSWs. Venue-based FSWs are women who are officially employed as hostesses to work in entertainment places (e.g. beer bars 'drinkshops', karaoke bars, nightclubs, guesthouses and restaurants) to provide services to clients in the form of conversation, serving beer and snacks, but also selling sex [7]. Venue-based FSWs provide sexual services outside the entertainment establishments in guesthouses or hotels.

A behavioral survey from 2009 among 912 FSWs in Laos found that 31% reported that they had experienced STI symptoms in the past three months [7]. The second HIV surveillance and STIs periodic prevalence survey in 2004 showed that 43 and 61% of FSWs had reported STI symptoms in the past 12 months in Vientiane (the capital of Laos) and in Savannakhet province, respectively. The FSWs also seemed to lack knowledge about STIs and many reported that they had received insufficient information related to STIs including HIV [2].

Access to and availability of good quality STI services is still relatively limited in Laos, especially in rural areas. In a study from Laos, HCPs, especially in rural areas, reported inadequate knowledge and competencies regarding management of reproductive tract infections (RTIs), including STIs [8]. Owing to the lack of STI clinics and clinical STI specialists, patients with STIs are mainly seen by gynecologists or general health practitioners, often with low competencies in STI management [8]. In addition, self-medication for RTIs including STIs through private pharmacies is common among FSWs in Laos [7]. Results from another study suggest that the main barriers to seeking health care among patients with RTI/STI symptoms were both structural (e.g. travel costs, clinic opening hours, and social stigma) and individual (e.g. fear of social discrimination and clinicians' negative attitudes) [9]. Seeking testing and treatment for STIs is also hampered by negative attitudes of HCPs, particularly for FSWs [10]. In Vietnam, FSWs seek treatment from private pharmacies and their decision to seek care is compromised by high costs, long waiting time, and judgmental attitudes of HCPs [11].

There are thus complex aspects that prevent FSWs from seeking healthcare services. How FSWs in Laos utilize public and private healthcare services has received insufficient attention and is not well documented. Understanding how FSWs seek care for their genital symptoms and the factors that influence their care seeking will provide policy makers with information needed to design appropriate interventions for FSWs. The aim of this study is to describe care seeking behaviour and barriers to accessing RTI/STI services and to describe the associations between background characteristics and care seeking behaviour among FSWs in Laos.

## Methods

### Study area

Savannakhet province, with a population of 826,000, is located 550 Km south of Vientiane. There are 15 districts with one provincial and 14 district hospitals, 94 health centres, 34 private clinics, one public pharmacy in each hospital, and 155 licensed private pharmacies in the province. In each district there are between 2 and 12 health centres and a number of pharmacies. Training in STI syndromic case management was implemented in this province in 1999 and afterwards additional STI training has occasionally been conducted. The training has focused on health personnel from the 15 hospitals, mainly those working in gynecology-obstetric wards. Health personnel from private clinics and private pharmacies have also been trained [12]. In 2003, a 100% condom use program and provision of antiretroviral treatment (ART) for HIV positive persons was introduced.

In 2006, a drop-in centre was established in Kaysone Phomvihan, the main district of the province. The centre is under supervision by the Secretariat of HIV/AIDS/STI Prevention and Control Unit, Savannakhet Province. Activities are designated for high risk groups such as FSWs, migrant workers and those requesting anonymous tests. The bar owners or pimps in the target districts are involved in providing health information and supporting FSWs to have regular check-ups for STI and to visit health facilities when ill. In 2008, a voluntary counseling and testing (VCT) program for HIV was put into place and 21,185 persons have been tested so far. The cumulative number of HIV positive persons in Savannakhet is 1,114, and the majority of those are FSWs and migrant workers. Behaviour change communication (BCC), advocacy for condom use, condom distribution, and RTI/STI treatment are also free of charge at the drop-in centre and has reached about two-thirds of the estimated 450 FSWs in the province [12]. However, if medication is limited or not available the patients will get a prescription and be requested to buy the recommended medicines in a pharmacy using their own money.

The centre also provides treatment services at district hospitals along Road 9, involving health personnel from gynecology-obstetric wards in order to achieve better coverage of services for FSWs as well as the general population. Recently, the drop-in centre has extended their services and now collaborates with about two-thirds of the district hospitals in the province. At health centres, healthcare services are mainly immunization and antenatal care. Treatment services for STIs are rarely available at health centres and most STI cases are referred to the drop-in centre, which is integrated within the district hospital. From the different entertainment places, it takes between 10 and 40 min by bicycle to reach a health centre or a drop-in centre.

Despite these efforts, the province has, in the Lao context, a high rate of STIs (33% for Chlamydia and 18% for gonorrhoea) and HIV (3.9%) among FSWs [2], and a large number of people are living with HIV/AIDS [13]. Savannakhet is the most HIV-affected province in country. The province was selected as study site because there are many entertainment places where FSWs work and live along Road 9, a main route, which runs across the province connecting Thailand with Vietnam (East-west corridor). In 2007, this province had 51 entertainment places and approximately 350 FSWs. In 2008, this had increased to 82 entertainment places and about 450 FSWs [12]. There are only venue-based FSWs in Savannakhet province.

### Study design and participants

This cross-sectional study was performed from January to March 2010 in six districts located along Road 9: Kaysone Phomvihan, Outhoumphon, Atsphangthong, Phalanxay, Phin, and Xepon in Savannakhet province. The study participants were women working in the entertainment places in six districts at the time of the interview. The inclusion criteria were women who: 1) were willing to participate; 2) were able to communicate in Lao and 3) self-reported selling sex according to the study's screening question.

### Data collection procedure

Six female social workers were recruited as interviewers. They were trained for three days on the aim of the study, data collection procedures and ethical issues. The survey questionnaire had closed and open-ended questions and consisted of four sections. The demographic section included questions on age, education, duration of sex work, place of work, and monthly income. In the reproductive health section, information about occurrence and duration of genital clinical signs or symptoms of RTI/STI was obtained. In the source of treatment and care seeking behaviour section, open-ended questions about care seeking behaviour and reasons for not seeking care when having RTI/STI symptoms as well as perceptions about services when seeking care for RTI/STI symptoms were included. Examples of such questions are: time from first symptom onset to first visit to a health facility, seeking care or not for RTI/STI symptoms during the previous 3 months, source of RTI/STI treatment at last visit, and experiences of and barriers to health seeking. The final section included questions about source of RTI/STI information. Spontaneous comments by the respondents were also registered.

A pilot study was conducted with 21 FSWs in a district not included in the study setting in order to test the questionnaire. Minor modifications of the questionnaire were made after the pilot in order to ensure that the words used were understandable and acceptable. The results from this pilot study are not included in the analysis.

Prior to data collection, the interviewers conducted a mapping procedure to identify entertainment establishments and FSWs in the study area. The names of FSWs and the number and type of entertainment establishments in the six districts were listed. We aimed to interview all FSWs in the study area. In order to prevent a repeat interview, since FSWs often change location, a screening question on whether she had been interviewed within one month was asked to each respondent prior to the interview. Moreover, respondents' names were identified and compared to the information from the mapping in each entertainment place.

**Figure 1:**
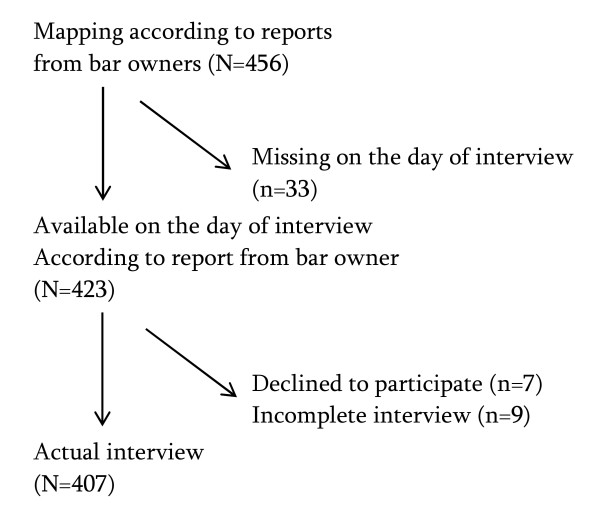
**Flowchart of number of FSWs who were approached, who declined and who were interviewed**.

In total, 456 FSWs from 76 entertainment places were identified during the mapping procedure and all of the women identified sold sex. On the day of the interview 33 of those were not available. In addition, seven declined to participate and nine first accepted to be interviewed but later refused to complete the interview without giving any reason. The remaining 407 (96%) were included in the analysis (Figure 1). Face to face interviews were performed in a place where respondents felt relaxed and confident to openly discuss the interview questions, mostly behind the bar in their working place. The interviews lasted approximately 35-40 min.

### Analysis

Data were double entered into Epidata 3.0 (Epidata Association, Denmark) and consistency checks were run to inspect the validity of the two data sets. Incorrect entries were examined and verified against the original forms. Data were analyzed using STATA v.10 (Stata Corp 2002; College Station, Texas, USA). The outcome measures, care seeking behaviour was analyzed and presented with basic descriptive statistics (frequencies, mean, median and standard deviation). The responses from the open-ended questions, barriers in accessing healthcare services, were coded and grouped into categories according to content (barriers for those who had and had not sought care). Multivariate logistic regression analysis was applied to identify associations between care seeking behaviour as dependent variable (seeking or not seeking care) and respondent's characteristics as independent variables (age group, education, monthly income, place of work, and duration of sex work). Odds ratios [OR] with 95% confidence intervals (95%CI) were used to measure associations. A p-value of 0.05 or less was considered as indicating a statistically significant association.

### Ethical considerations

Ethical clearance was received from the National Ethics Committee for Health Research, Laos and from the Regional Ethics Committee in Stockholm, Sweden. Permission to carry out the study was also obtained from the local authority where the study was implemented. Verbal consent was sought from each respondent and the permission to interview was also obtained from the bar owner or the pimp. The participants were ensured that their responses would be treated in confidence. Moreover, they were assured anonymity and were informed that they were free to refuse or end the interview at any time without giving any reason. The respondents were offered condoms and STI information after the interview.

## Results

### Background characteristics

The mean age of the respondents was 20 (range 15-31). About half (49%) were adolescents (aged 15-19). Forty three percent had attained primary school level, whereas 50% reported that they had started or completed secondary school. The median income of the respondents was 300 USD/month (1 USD equivalence 8.340 Lao kip, March 2010). The majority of the respondents (78%) worked in a beer bar. About two-thirds had engaged in sex work less than one year. The profile of respondents is shown in Table 1.

**Table 1:** Background characteristics of the respondents (n = 407)

Characteristics		n = 407	%
Age group (years)	Mean: 20; range: 15-31		
15-19		199	48.9
20-24		160	39.3
25-31		48	11.8
Education			
No formal school		27	6.6
Primary school		176	43.2
Secondary or high school		204	50.1
Monthly income (USD)	Mean: 300; range: 48-900		
< 300		238	58.5
300-600		137	33.7
> 600		32	7.9
Work place			
Beer bar FSW		318	78.1
Guest house FSW		53	13.0
Nightclub/restaurant FSW		36	8.9
Duration of sex work (years)	Mean: 1.5; range: 0.1-6		
< 1		238	58.5
1-2		93	22.9
>2		76	18.7

### Reproductive tract conditions

Respondents' reproductive tract conditions are shown in Table 2. The majority of respondents (86.7%) reported having RTI/STI symptoms in the past three months. About 41% had experienced RTI/STI symptoms for four weeks or more. Among respondents who reported current RTI/STI symptoms, 76.2% reported having abnormal discharge, 56.3% lower abdominal pain, and 50.9% genital itching.

**Table 2:** Respondents' reproductive tract condition (n = 407)

Variable		n = 407	%
RTI/STI sign/symptoms currently or in last 3 months			
Yes		353	86.7
No		54	13.3
Length of time having RTI/STI symptoms	(n = 353)		
Median: 4 weeks;	(range: 2-14 weeks)		
< 4		206	58.4
4-8		98	27.7
> 8		49	13.9
Current RTI/STI sign or symptoms*	(n = 316)		
Discharge		310	76.2
Lower abdominal pain		229	56.3
Genital itching		207	50.9
Pain on intercourse		137	33.7
Pain on urination		90	22.1
Genital wart/ulcer		53	13.0

*Multiple responses were allowed; the sums of responses are therefore greater than 100%

### Care seeking behaviour for RTI/STI symptoms

Respondents' care seeking behaviours are presented in Table 3. About two-thirds of the respondents had sought care for their genital symptoms in the past three months, whereas one third had not. Of those respondents who had sought care, 58% delayed less than one week while 19% delayed more than two weeks. The mean time of delay from onset of symptom to first visit to a healthcare agency was ten days. Those who had sought care reported using a range of different healthcare agencies. About 53% had visited the drop-in centre, 24% had sought care from a public hospital, and 12% from a private clinic. About one in ten had sought care from a pharmacy/drugstore and only 5 persons sought care from a herbalist. Of the respondents with symptoms who had not sought care, 62% were 15 to 19 years old (data not shown).

**Table 3:** Care seeking behaviour among respondents RTI/STI symptomatic (n = 353)

RTI/STI symptomatic currently or within last 3 months		n = 353	%
Sought care for RTI/STI symptoms			
Yes		238	67.4
No		115	32.6
Time delay until seeking care (days)	(n = 238)		
Mean: 10.2	(range: 2-60)		
< 7		137	57.6
7-14		55	23.1
> 15		46	19.3
Source of RTI/STI treatment the last time	(n = 238)		
Drop-in centre		126	52.9
Public hospital		56	23.5
Private clinic		29	12.2
Pharmacy/drugstore		22	9.2
Herbalist		5	2.1

### Barriers to and perceptions about RTI/STI services

Although 238 respondents had sought care for RTI/STI symptoms, 38% (n = 90) still reported barriers to accessing the services, mainly long clinic waiting time (67%) and inconvenient location of the clinic (31%). Judgmental attitudes of HCPs and very bureaucratic procedures to use the services were mentioned by ten and nine percent respectively. The main barriers mentioned by those who reported symptoms of RTI/STI (n = 115) in the last three months but had not sought care were inconvenient location of the clinic (50%), not knowing where they could get the service needed (25%), and long waiting time at the clinic (20%). In addition, lack of money and lack of time to visit the clinic were reported by almost one-fifth of those who did not seek STI treatment (Table 4). Some respondents provided spontaneous comments about HCPs' attitudes, reporting that they 'look down on me', 'behave badly towards me', and 'present an unwilling attitude'.

**Table 4:** Barriers to RTI/STI services mentioned by respondents

	Sought treatment for RTI/STI					
Barriers	Yes		No		Total	
	n = 90	%	n = 155	%	n = 245	%
Long waiting time	60	66.7	23	20.0	83	32.7
Inconvenient location	28	31.1	57	49.6	85	33.5
Judgmental attitude	9	10.0	20	17.4	29	11.4
Bureaucratic procedures	8	8.9	8	7.0	16	6.3
Lack of money	6	6.7	22	19.1	28	11.0
Don't know where to go	4	4.4	29	25.2	33	13.0
Don't have time	4	4.4	22	19.1	26	10.2
Lack of confidentiality	0	0	19	16.5	19	7.5
Don't care not important	0	0	16	13.9	16	6.3
Afraid of the result	0	0	1	0.9	1	0.4

* Multiple responses were allowed; the sums of responses are therefore greater than 100%

### Source of information regarding STIs

Table 5 shows sources of health information on STIs mentioned by respondents who had or had not sought care for RTI/STI symptoms in the previous three months. The main source of information among those who had not sought care was their fellow friends (50%), peer educators (37%) and pimps (36%), whereas respondents who had sought care received information from peer educators (63%), HCPs when visiting a healthcare agency (40%), their fellow friends (33%) and pimps (27%). Very few respondents had received information from the media.

**Table 5:** The main source of information regarding STI

	Sought treatment for RTI/STI					
Main source of information*	Yes		No		Total	
	n = 238	%	n = 115	%	n = 407	%
Peer educator	151	63.4	43	37.4	194	43.7
Fellow friends	79	33.2	58	50.4	137	33.7
Pimp	63	26.5	41	35.7	104	25.6
HCP during the visit	95	39.9	11	9.6 4.3	106	26.0
Boy friends	13	5.5 0.8	5	4.3	18	4.4
TV	2	0	5	0.9	7	1.7
Radio	0		1		1	0.2

*Multiple responses were allowed; the sums of responses are therefore greater than 100%

### Predictors of care seeking behaviour

The result of the multivariable logistic regression analysis is presented in Table 6. Duration of sex work was significantly associated with seeking RTI/STI treatment. Respondents who had worked as sex workers for one year or more were more likely to seek treatment compared to those who were new in sex work (OR: 2.6; 95%CI: 1.52-5.36, *p *= 0.001). Other background characteristics such as education, monthly income and work place were not significantly associated with seeking care.

**Table 6:** Association between respondents characteristics and care seeking behaviours (n = 407)

Variables	Crude OR*	P-value	Adjusted OR	P-value
	(95% CI)**		(95% CI)	
Age group (years)				
15-19	1		1	
20-24	1.31 (0.81-2.13)	0.259	1.13 (0.68-1.88)	0.630
25-31	1.04 (0.50-2.14)	0.910	0.91 (0.41-1.99)	0.891
Education				
No formal school	1		1	
Primary	1.68 (0.68-4.15)	0.259	2.20 (0.85-5.73)	0.103
Secondary and high school	1.89 (0.77-4.65)	0.162	2.21 (0.85-5.71)	0.101
Monthly income (USD)				
< 300	1		1	
300-600	1.19 (0.73-1.93)	0.478	1.12 (0.67-1.88)	0.643
> 600	2.14 (0.77-5.94)	0.143	1.66 (0.54-5.09)	0.372
Work place				
Beer bar/drink shop	1		1	
Nightclub	1.11 (0.50-2.45)	0.781	0.93 (0.37-2.31)	0.879
Guest house/Karaoke	1.39 (0.68-2.83)	0.353	0.99 (0.43-2.25)	0.987
Duration of sex work (years)				
< 1	1		1	
1-2	2.73 (1.49-4.99)	0.001	2.68 (1.52-5.36)	0.001
>2	2.01 (1.09-3.68)	0.024	1.80 (0.87-4.01)	0.109

*OR: odd ratio; **95% CI: 95% confidence interval

## Discussion

We found that FSWs faced several challenges in accessing healthcare services. Eighty-seven percent of the respondents reported RTI/STI signs or symptoms currently or in the last three months. However, only two thirds of the symptomatic respondents sought care. This is an alarming result because delayed treatment of STIs could have a profound impact on the health of the women both during their time as sex worker and in their future lives. Delay of or mistreatment for STIs has been shown to result in infertility, ectopic pregnancy, cervical cancer [14], and increased risk of acquiring HIV [15,16]. We found that two-thirds of those who did not seek treatment for RTI/STI symptoms were adolescents. This group is particularly at risk of STI and HIV infection due to immaturity of the genital tract, less power to negotiate condom use, and higher number of sexual clients compared to their older peers as has been shown in other settings [17,18]. Furthermore, adolescent FSWs often lack knowledge about the consequences of unsafe sex [19,20]. In addition, a study in Thailand found that minors in sex work reported a higher proportion of sexual violence compared to those who were older [21].

Of the respondents who had sought RTI/STI treatment in the previous three months, slightly more than half had visited a drop-in centre and about one-fourth had visited a public hospital. We don't know, however, if the public hospitals that the respondents visited were also drop-in centres, since such centres have been established within public hospitals. A concern is that the establishment of a centre for FSWs within a public hospital could influence FSWs health seeking because they do not want to be seen by other patients. In order to prevent this, the drop-in centre could be located outside the district hospital and the name phrased neutrally in order to avoid negative perceptions. Lessons learned from another setting suggest that confidentiality and privacy in STI treatment services is an essential and effective method for individual compliance to treatment, as it significantly increased STI patients' attendance [22]. About one-fifth of respondents visited private clinics or pharmacies. This is an important concern in Laos where there are no designated STI clinics, and pharmacists/drug sellers are a common source of medical advice for STI patients [9]. Another study from Laos found that drugs obtained from pharmacies often have limited efficacy in curing STIs [23]. Moreover, pharmacists/drug sellers have been found to have inadequate knowledge and competence in the management of STI [8,10]. STI training for STI service delivery staff from both the public and the private health sectors is needed. Barriers mentioned by respondents who had sought care were mainly related to quality of care. Future studies on these topics are recommended in order to improve the existing services in this particular setting.

The majority of the respondents who had not sought care for RTI/STI symptoms in the previous three months cited barriers such as inconvenient location of the clinic but also that they did not know where to get treatment. Long waiting times at the clinic, lack of time and money, lack of confidentiality and judgmental attitudes of HCPs were also mentioned as barriers. Although stigma was not explicitly mentioned by the respondents, their spontaneous comments about providers' judgmental attitudes could be perceived as stigmatizing. Furthermore, the words for judgmental attitudes and for stigma are similar in the Lao language. Those who had not sought care mentioned barriers that presumably resulted from previous negative experiences when using healthcare services such as long waiting times at the clinic and inconvenient location.

Long waiting times at the clinic may encourage FSWs to leave the clinic without being seen by a HCP, as has been reported elsewhere [24]. A study in the United Kingdom found that "structural" barriers to FSWs' use of health services were lack of information about the available services and inconvenient opening hours of the clinic [25]. Individual barriers identified in other settings are fear of being judged, high cost of services and difficulties getting to the clinic [26,27]. Our findings suggest that more attention should be paid to those respondents who claimed that they did not know where they could get the services they needed, with a focus on those who are just entering sex work. This indicates the need to upgrade the existing information and find creative ways of communicating this information to FSWs, particularly adolescents who are at greatest risk according to our results.

The respondents who had not sought care received health information mainly from fellow friends but also from peer educators and pimps. FSWs are a mobile population, so delivering health information and providing them with STI services is a challenge. Establishment owners or pimps should be trained and involved in the delivery of health information. Peer referral approaches, which have proven to increase clinic attendance in other settings [28,29], should continue and the drop-in centre approach designed in an appropriate and confidential manner should be expanded to more districts of the province. The one factor that was associated with less care seeking in our study was shorter duration of sex work. This could partly be explained by the fact that FSWs are more aware of risks related to their work and learn to deal with their health problems after a period of time. This implies that those who are just beginning to work as FSWs are particularly in need of information about where to access services and the importance of doing so. It also reflects the vulnerability of younger FSWs.

Timely detection, correct diagnosis and appropriate treatment are essential factors for quality STI services [22]. Despite STI services being available and accessible in the study districts, which are urban areas, we found that most respondents did not seek care as soon as they experienced the symptoms, with over two-thirds having symptoms for four weeks or more. Such delays might lead to further transmission of STI, increased drug resistance and severe consequences of STIs [30]. Despite public facilities offering services free of charge to patients, a higher proportion of those who had not sought care mentioned lack of money as a barrier for seeking care compared to those who had sought care. We assume that some FSWs did not have information that the available services were free of charge, especially those who were new in sex work. However, the concerns about cost that FSWs mentioned could have been due to indirect costs such as travel costs or the cost of buying drugs when the recommended drugs were not available in the clinic.

Although Laos has a national policy and action plan in place, the management of RTIs/STIs for FSWs still faces several challenges. Although HCPs are seen as the basic source of treatment and information, we found a large proportion of respondents who said that they did not receive health information from HCPs during the visit. Health information delivered through HCPs may have difficulty reaching these women. This was also indicated in a previous study in the same district where HCPs had negative attitudes towards FSWs with STI symptoms, with many agreeing with the statement: "even if FSWs are counselled, they would continue to infect others" [10]. Innovative methods of providing STI information is needed in order to enhance women's access to the information they need. Establishment of hot line service centres providing information via a mobile phone might be considered. Furthermore, STI treatment by a mobile team for FSWs could be piloted in this particular setting.

## Limitations

Using female interviewers with a non-medical background was a suitable methodology for data collection in this study. Mapping helped to identify FSWs in the study setting and helped avoid potential bias from repeated interviews with the same FSW. Furthermore, each interview was conducted in a location convenient to the participants where a conversation could be held in privacy so the women could feel free to discuss their personal experiences. However, this study has limitations. Data were only collected from women who were available the day of interview. The views of those who were absent seeking treatment or attending to clients were thus not covered. We also do not know if those who visited hospitals did so because there was a drop-in centre located there. Despite the limitations, this study provided impetus for future research and valuable information that can be used when designing services and information campaigns for FSWs in this particular setting.

## Conclusions

FSWs sought care from both public and private health facilities such as drop-in centres, public hospitals, private clinics and private pharmacies/drugstores. There were several barriers to accessing RTI/STI services and they were related to both structural and individual factors including inconvenient location, long waiting time in the clinic, not knowing where to go and judgemental attitudes of healthcare providers. FSWs delayed visiting a health care centre by on average of ten days.

## Recommendations

Innovative STI service strategies to inform FSWs about the importance of early diagnosis and treatment should be designed. To increase clinic attendance among FSWs, the centre should provide patients an environment that respects their privacy and have notices to inform patients that they can ask for privacy anytime they feel inconvenienced. The clinic waiting time should be minimized by developing operational guidelines to ensure that patients who require urgent medical attention are prioritized. In order to improve the quality of the available STI services, continuous training for STI service providers with emphasis on counseling skills and awareness of the sexual healthcare needs for FSWs is recommended.

## Competing interests

The authors declare that they have no competing interests.

## Authors' contributions

The main author (KP) developed the research design, prepared data collection, supervised RAs during data collection, carried out the analysis, and drafted the manuscript. EF, ST and VS assisted with the research design and offered critical comments in the reviewing and writing of the manuscript. All authors have read and approved the final version of the manuscript.

## Pre-publication history

The pre-publication history for this paper can be accessed here:

http://www.biomedcentral.com/1472-6963/12/37/prepub
